# Psychological assessment in school contexts: ethical issues and practical guidelines

**DOI:** 10.1186/s41155-024-00318-x

**Published:** 2024-08-20

**Authors:** Irene Cadime, Sofia A. Mendes

**Affiliations:** 1 Centro de Investigação em Estudos da Criança, Universidade do Minho, Braga, Portugal; 2 Centro de Investigação em Psicologia para o Desenvolvimento, Instituto de Psicologia e Ciências da Educação, Universidade Lusíada Porto, Porto, Portugal

**Keywords:** School psychology, Psychological assessment, Ethical issues

## Abstract

**Background:**

Psychological assessment in school settings involves a range of complexities and ethical dilemmas that practitioners must navigate carefully. This paper provides a comprehensive review of common issues faced by school psychologists during assessments, discussing best practices and ethical guidelines based on codes from various professional organizations.

**Methods:**

We examine the entire assessment process, from pre-assessment considerations like informed consent and instrument selection to post-assessment practices involving results communication and confidentiality. Key ethical concerns addressed include fairness in assessment, cultural and linguistic appropriateness of testing materials, and issues surrounding informed consent.

**Results:**

Specific challenges discussed include selecting appropriate assessment instruments that reflect the diverse needs and backgrounds of students, ensuring fairness and removing bias in testing, and effectively communicating results to various stakeholders while maintaining confidentiality. We emphasize the importance of multi-source, multi-method assessment approaches and the critical role of ongoing professional development in ethical practice.

**Conclusion:**

By adhering to established ethical standards and best practices, school psychologists can effectively support the educational and developmental needs of students. This paper outlines actionable recommendations and ethical considerations to help practitioners enhance the accuracy, fairness, and impact of their assessments in educational settings.

## Introduction

Psychological assessment is central in the work of school psychologists. They can use assessment for the purpose of obtaining a diagnosis, but most of the time assessment is used for purposes of eligibility determination (for example, for special education services) and to make data-based decisions (Benson et al., 2019; Seabra-Santos et al., 2019). This includes using the assessment to inform evidence-based intervention programs and instructional strategies that are intended to improve students’ achievement and well-being as well as using it to flag students at risk and to monitor progress in a multitiered system of support (Braden, 2013; Hendricker et al., 2023; Truckenmiller & Brehmer, 2021).

According to the European Federation of Psychologists Association (EFPA) Board of Assessment, psychological assessment is a “systematic method or procedure for ascertaining the psychological characteristics of an individual or group of individuals, or the performance of an individual or group of individuals” (EFPA, 2023a, p. 2). In this paper, we use the term psychological assessment broadly, assuming that it can cover a wide range of psychological functions, including cognitive abilities, personality traits, emotional functioning, and behavioral patterns, although it can be argued that most of the assessment conducted by school psychologists is, in fact, psychoeducational assessment which is primarily aimed at understanding an individual’s learning profile, academic strengths and weaknesses, and educational needs (Lovett et al., 2022; Wodrich et al., 2006). In Portugal, school psychologists extend this scope by conducting psychological assessments that not only address academic purposes but also include career guidance and evaluations requested by child protection agencies, courts, healthcare services, and community institutions (Mendes et al., 2018).

Regardless of the focus, a multitude of assessment methods, including standardized tests, interviews, and direct observations, can be used to collect data and develop hypotheses about the psychological characteristics or performance of an individual or group (Maluf et al., 2022). This way, psychological assessment in schools has methodological similarities with other contexts but also has specificities. The first specificity is that most of their work focuses on children and adolescents, but it also includes collaboration with parents, teachers, and a variety of other school staff as well as professional and community services that interact with children and adolescents (Mendes et al., 2014).

In several countries, including Portugal, schools are receiving an increasing number of refugee and migrant children who frequently experience difficulties of adaptation and low academic achievement (Guedes et al., 2021; Seabra & Mateus, 2020). This poses an additional challenge for school psychologists, as some of these children do not master the native language and most of the available assessment instruments are not validated for such diverse populations. Additionally, schools (mainly the public ones) receive a diversity of students, including students with disabilities and from poor backgrounds (Braden, 2013; Couto et al., 2021). Thus, psychological assessment in school contexts sets several challenges and ethical issues that practitioners must consider.

In this paper, we explore the frequent challenges encountered in school assessments and highlight recommendations from ethical codes to address these issues. Over the last decade, there has been a significant increase in the number of psychologists working in Portuguese schools, which underscores the importance of effective assessment practices. For instance, the ratio of psychologists to students in public schools improved from 1:1311 in 2012 (Mendes, 2019), to 1:744 in 2020 (CNE - Conselho Nacional de Educação, 2022). To support this growing presence of psychologists in schools, a framework for school psychologists was recently published by the Portuguese General Directorate of Education and the Order of Portuguese Psychologists (Breia et al., 2024). This framework covers various assessment methods, including tests, interviews, and observations, and offers insights into the content and delivery of reports. Despite these advancements, the framework does not deeply delve into the ethical issues surrounding assessment practices, highlighting a gap that needs further exploration to ensure assessments are conducted ethically and effectively. Therefore, there is an increasing need for updated and comprehensive information to guide psychologists’ work in psychological assessments in schools, considering the expanded framework of international standards. This paper focuses particularly on the professional standards of the National Association for School Psychologists (NASP, 2020) in the United States, the European framework of standards for educational assessment published by the Association of Educational Assessment – Europe (AEA, n.d.), and the international guidelines for test use by the International Test Commission (ITC, 2013). Additionally, we refer to the ethics codes of the Order of the Portuguese Psychologists (OPP, 2021) (currently under review) and the International School Psychology Association (ISPA, 2021). For the sake of clarity, we organize this review into two sections, dividing it into challenges and dilemmas that occur before and after the administration of assessment techniques, although we recognize that the assessment process does not begin or end with the administration of these.

## Before the administration of the assessment techniques

Psychological assessment starts well before the administration of tests, interviews, or observation grids. It is not our goal to deep dive into the steps of psychological assessment, but in general, psychologists should first understand why the assessment is being requested and who requested it (e.g., parents, teachers, other professionals or even the student himself/herself) as well as gather initial information about the student’s medical, educational, family, and social history (Schneider, 2014). This will allow the psychologist to set goals for the assessment and to develop working hypotheses for the case (Fernández-Ballesteros, 1996). The next steps are to select the assessment instruments, to guarantee fairness in assessment, and to collect informed consent. Next, we present some of the most common challenges for school psychologists during these stages.

### How to select the assessment instruments?

The selection of assessment instruments is the first task of school psychologists after determining the need of a psychological assessment and its goals. The first aspect that school psychologists must take into account is that relying on a single assessment source may provide an incomplete picture of a student’s abilities and needs. Ethical practice involves considering information from multiple sources, such as teachers, parents, and other relevant individuals, to make well-informed decisions (Benson et al., 2019; Genachowski et al., 2023). Thus, school psychologists should strive to use multisource, multimethod, and multifactored assessments as much as possible (Riccio & Rodriguez, 2007).

Additionally, school psychologists should use assessment techniques that are valid for the student and for the goal of assessment (NASP, 2020). This last aspect is of utmost importance, as the purpose of the assessment and the hypotheses about the case are key to instrument selection. Ethical conduct implies avoiding the administration of testing protocols “one size fits all” to all children and adolescents seeking or being referenced to the school psychologist services, regardless of the motive. Table 1 summarizes some of the questions that psychologists should take into account when selecting standardized tests for psychological assessment as well as some of the most relevant standards regarding each one. When doing this selection, the psychologist should consider whether the test not only allows them to meet the goals of the assessment but also has sound psychometric properties, such as reliability and validity.

**Table 1:** Questions when selecting standardized tests for psychological assessment and relevant international standards/guidelines

Question	Relevant standards/guidelines		
	NASP Professional Standards (2020)	AEA European Framework of Standards for Educational Assessment (n.d.)	ITC Guidelines for Test Use (2013)
**Does the test meet my specific assessment goals and objectives?**	Standard II.3.2 Assessment Techniques	1.5.1 Defining the goal (construct, target group, function) 1.5.2 Identifying the nature of evidence and tasks	2.1. Evaluate the potential utility of testing in an assessment situation
**Is the test appropriate for my target population (e.g., age, developmental stage, cultural, linguistic, and socioeconomic background)?**	Standard II.3.4 Normative Data	1.5.2 Identifying the nature of evidence and tasks 1.5.5 Decision making (aggregating, norms, grades, cut off scores)	2.2. Choose technically sound tests appropriate for the situation
**Are there accommodations available for non-native speakers or individuals with disabilities?**	Standard II.3.8 Validity and Fairness		
**Is there strong evidence that the test is valid (i.e., measures what it intends to) and produces reliable, consistent scores over time?**	Standard II.3.3 Instrument Selection	1.5.2 Identifying the nature of evidence and tasks 1.5.7 Evaluation and next iteration	2.2. Choose technically sound tests appropriate for the situation
**Does the test have up-to-date normative data for my target population?**	Standard II.3.4 Normative Data	1.5.5 Decision making (aggregating, norms, grades, cut off scores)	2.2. Choose technically sound tests appropriate for the situation 2.9. Review the appropriateness of the test and its use
**Is the test free from cultural, gender, or socioeconomic bias?**	Standard II.3.8 Validity and Fairness	1.5.2 Identifying the nature of evidence and tasks	2.3. Give due consideration to issues of fairness in testing
**Is the test practical in terms of accessibility, cost, and the resources needed (e.g., time, materials, technology)?**	Standard II.3.3 Instrument Selection Standard II.3.5 Digital Administration and Scoring	1.5.2 Identifying the nature of evidence and tasks 1.5.3 Gathering evidence (admin and logistics) 1.5.7 Evaluation and next iteration	2.2. Choose technically sound tests appropriate for the situation
**Do I have the necessary competence to administer and interpret the test, or is additional training required?**	Standard II.1.1 Practice in Area of Competence	1.5.2 Identifying the nature of evidence and tasks 1.5.3 Gathering evidence (admin and logistics) 1.5.4 Capturing outcomes (scoring, rating)	1.2. Ensure they have the competence to use tests
**Are there clear procedures for scoring and interpreting test results?**	Standard II.3.11 Interpretation of Results Standard II.3.5 Digital Administration and Scoring	1.5.2 Identifying the nature of evidence and tasks 1.5.4 Capturing outcomes (scoring, rating) 1.5.6 Interpreting and reporting results	2.6. Score and analyze test results accurately 2.7. Interpret results appropriately
**Does the test produce results that are easy to communicate to stakeholders (e.g., students, parents, educators)?**	Standard II.3.11 Interpretation of Results	1.5.6 Interpreting and reporting results	2.8. Communicate the results clearly and accurately to relevant others
**What are the potential consequences of the test results on individuals and the institution?**	Standard II.3.1 Considerations Prior to Disability Determination	1.5.7 Evaluation and next iteration	
**What are the pros and cons of using this test compared to others available?**	Standard II.3.6 Variety of Sources of Data	1.5.7 Evaluation and next iteration	2.1. Evaluate the potential utility of testing in an assessment situation
**Have I considered my own biases or assumptions in choosing this test?**	Standard II.3.8 Validity and Fairness		
**Does the test facilitate interdisciplinary collaboration for comprehensive assessment?**	Standard II.3.7 Comprehensive Assessment	1.5.7 Evaluation and next iteration	

A national survey conducted in 2017 in the United States about the assessment practices by school psychologists indicated that those used more standardized tests with robust psychometric properties, compared to previous decades, when projective tests, which had weaker evidence of reliability and validity, were quite frequently used (Benson et al., 2019). The almost nonexistent use of projective tests in the assessment conducted in schools in different countries was also highlighted in in a recent systematic review of the literature (Maluf et al., 2022). The same finding was recently reported in a study with Portuguese psychologists (Simões et al., 2024). Another important aspect that school psychologists should consider when selecting tests is utility. According to Canivez (2019), this can include “diagnostic utility (the correct identification of those who truly have a condition and those who truly do not have a condition) and treatment utility (that the assessment information resulted in recommendation of a specific treatment that as a result improved the client’s functioning)” (p. 196).

Whether using standardized tests or other assessment methods such as interviews, psychologists must be qualified to administer and interpret the chosen assessment instruments, as the lack of expertise can lead to inaccurate results, misinterpretation of data, and inappropriate interventions. Thus, the training for the administration of the assessments is critical.

The guidelines from the EFPA (2023b) indicate that, in the first cycle of the bachelor’s program, there should be a focus on providing students with a comprehensive theoretical foundation in assessment instruments, including psychometric principles, theoretical underpinnings, and test construction. In the second cycle, the emphasis should shift to hands-on practice, allowing students to develop practical competencies through supervised experiences in test administration, scoring, and interpretation, helping them apply their theoretical knowledge in real-world settings. In this regard, given the limited time during initial education and training, it might be most reasonable to focus on fewer instruments, allowing students more opportunities for practice in class.

In Portugal, there are no national standards for psychology training and it is unclear whether students are satisfied with the training they receive. Moreover, there is no consensus on a common set of assessment instruments to be taught across all psychology courses offered by different universities. The survey conducted by the EFPA Board of Assessment, in 2019/2020, suggested that psychologists in Italy and Croatia are dissatisfied with the training they received at the university level regarding testing (Lis et al., 2022). More studies are needed to understand whether this dissatisfaction also occurs in other countries and whether this is particularly true for school psychologists.

Additionally, even when a psychologist has received specific training and practice with an assessment instrument, it is unlikely that this professional has mastered the use of that instrument for all purposes and in all contexts where it might be used. For example, a school psychologist can have mastered the use of cognitive tests within the scope of determining eligibility for special education services but may be unable to use them to assess whether a child is a reliable witness in a criminal case and make a recommendation. This scenario highlights the need for school psychologists to develop competencies in specific areas of psychological assessment, as emphasized by the APA guidelines for psychological assessment and evaluation (APA, 2020). According to these guidelines, effective assessment requires more than just knowing how to administer and score tests:

a psychologist working in a school environment with a task of identifying children in need of special educational services not only strives to be competent in knowing how to select, administer, and interpret a psychological test of cognitive ability, academic achievement, or emotional adjustment and functioning but also seeks to know and understand special education law […], as well as the student’s cultural context, the classroom context, and how it affects manifestation of learning and adjustment difficulties. In addition, a suitable level of knowledge about best practices in classroom methods is important to make helpful and appropriate recommendations of educational interventions based on test data gleaned from the use of psychological tests (American Psychological Association, 2020, p. 13).

The constraints of the recent COVID-19 pandemic have also raised a renewed interest in online remote psychological assessment. However, several concerns have been raised about this modality of assessment, among which are as follows: the lack of normed standardized tests for remote administration; the lack of training for psychologists to perform remote assessments; the limitations in gathering important data such as behavioral observations (observations on screen may not be as rich and complete as face-to-face observations); the probable existence of technical issues, such as the quality of network connections and the availability of adequate devices and platforms; and, in the case of children’s assessments, the fact that the presence of an adult (most likely the parents) accompanying the child is needed to act as a facilitator, which may raise some conflicts of interest (Farmer et al., 2021; Vijayanand & Raman, 2022).

### How to foster fairness in assessment?

Ensuring that assessments are fair and unbiased for all students is crucial. Socioeconomic, cultural, and linguistic factors can influence test performance, and psychologists must strive to minimize these biases to provide an accurate representation of a student’s abilities. This requires ongoing monitoring and evaluation of assessment tools and procedures to identify and address any potential biases, inaccuracies, or shortcomings. In the case of standardized tests, one way of doing this is to prefer tests whose items have undergone differential functioning analysis (e.g., Borsa, 2016; Cadime et al., 2014). Differential item functioning analysis (DIF) is a statistical method used to identify whether different groups of test-takers (e.g., based on gender, race, or ethnicity) are being treated fairly by an assessment (Bialo & Li, 2022). DIF occurs when individuals from different groups with the same underlying ability level have a different probability of answering an item correctly (Zanon et al., 2016). During test development, when identified, those items can be reviewed and potentially revised or removed to ensure that they do not unfairly advantage or disadvantage any group. Thus, this helps to ensure that an assessment measures the intended construct equivalently across different groups, without bias or unfair advantage.

Tests are usually developed for an intended population (with demographic, linguistic, and cultural specificities) and for specific goals (AERA, 2014; AEA, n.d.). However, there are some situations in which psychologists have limited access to tests that fit the population and the purpose of the assessment and need to make use of other available instruments (Gilmore & Campbell, 2019). When the selection and use of an instrument deviate from its intended purpose and population—for example, using a cognitive test developed for preschool children to assess primary school children with learning disabilities or using a test whose norms were developed with children living in Portugal and applying it to children in Brazil—psychologists should acknowledge and communicate the limitations, potential biases, and errors that may arise from such deviations in assessments. Especially when using instruments outside their validated context, results should be interpreted cautiously (APA, 2020; ISPA, 2021).

When there is a substantial lack of standardized tests for a specific situation—for example, to assess a migrant child from a different country who speaks a different language—using alternative methods that typically provide qualitative information, while incorporating multiple sources of information, such as parent, student, and teacher interviews, observations, teacher reports, academic records, and modified assessments, can provide a more comprehensive and equitable evaluation of a student’s abilities and needs (Khawaja & Wotherspoon, 2022). Additionally, psychological assessments in schools can, in some cases, incorporate accommodations for student diversity, particularly for those with disabilities (e.g., deafness, blindness) and language differences. Standard testing conditions may not be equitable for all students, requiring adjustments such as alternative test formats, extended time, or language support (Thompson et al., 2018).

The International Test Commission (2013) provides guidance on when to make test accommodations for individuals with disabilities. If the disability is unlikely to affect test performance, or if it results in the loss of a skill that is integral to the construct being measured, no accommodations should be made. However, accommodations should be provided when the disability introduces irrelevant variance to the test scores. For example, accommodations, or even alternative measures, would be necessary if a student with vision loss has difficulty performing a working memory task based on visual stimuli. Different professional associations, such as the American Educational Research Association (2014), the National Association of School Psychologists (2020), and the American Psychological Association (2022) provide guidelines to ensure that assessments are fair, valid, and reliable when working with special populations. By adhering to these standards, school psychologists can provide a more accurate and equitable assessment experience for all students.

Another way of reducing bias in assessment is to adopt Multi-Tiered Systems of Support (MTTS) approach. The MTTS framework aims not only to identify students’ needs but also to adapt the educational setting, involving universal screening, evidence-based instruction, frequent progress monitoring, and increasingly intensive supplemental support and intervention for those students who do not respond positively to the instruction (Jimerson et al., 2016). Thus, this framework provides a set of low-inference assessment methods rather than relying on complex, high-inference standardized psychological assessments, such as cognitive ability tests, which often fail to effectively address the diversity of students in schools. In Portugal, this approach is relatively recent and has been progressively implemented in schools following the publication of legislation in 2018 (Decree-Law 54/2018).

### To whom and how to request the informed consent?

When the students are minors, obtaining informed consent from parents or legal guardians, in the form of a written agreement, is mandatory as indicated in ethical codes (ISPA, 2021; NASP, 2020; OPP, 2021). Informed consent agreements should include the reasons for and goals of the assessment, the procedures to be used, what the assessment results will be used for, and who will have access to the results (Knauss, 2001). However, ensuring that individuals truly understand the purpose, procedures, and potential consequences of the assessment can be challenging. School psychologists should explain these aspects to parents using a language and terms that they can understand. Some parents may be reluctant to consent, fearing the consequences of the assessment to their children. Efforts should be made to involve parents in the whole assessment process and to communicate openly and transparently with them about the results and implications for their child’s education and well-being.

The collection of students’ assent is also recommended, as this will probably foster their cooperation. When they understand what will happen and why it will be done, it is more likely that they will collaborate in the assessment process. Once again, it is important to explain this to students using language that they can understand. School psychologists should strive to obtain the assent, but it is not unusual for some students to still refuse to cooperate. In this case, as Knauss (2001) suggests “children who refuse to cooperate during individual testing are still usually evaluated using alternative measures such as observations or teacher and parent ratings” (p. 233).

## After the data collection: report and communication of results

After conducting the assessment, the next task is communicating the results. It is crucial to provide clear and understandable feedback to parents, guardians, and students. This involves explaining the assessment results and presenting recommendations along with potential implications for educational planning. However, several issues arise regarding the communication of results, primarily concerning the questions, “To whom should the results be communicated?” and “How to communicate the results?”.

### To whom should the results be communicated?

Research in various countries, including Portugal, shows that privacy and confidentiality issues are among the main ethical dilemmas faced by school psychologists (Dailor & Jacob, 2011; Jacob-Timm, 1999; Maki et al., 2024; Mendes et al., 2016). The practice in school contexts has particular characteristics that make the confidentiality of results particularly tricky. Historically, in psychology, the ethical dilemma to whom to communicate the results has been based on the question “who is the client?” (Fisher, 2014; Pantaleno, 1983). However, in school psychology, the client is frequently hard to identify. Most of the time, the school psychologist’s services concern children or adolescents, who would easily be identified as “clients”. However, in school settings, other professionals, such as teachers, special education staff, and administrators, may request information regarding the assessment results in order to inform educational decisions. Thus, ethical practice involves sharing information responsibly to support the learning and overall well-being of the student.

As such, it is hard to identify one sole client, as the students, their parents or legal tutors/guardians, and school staff, all could be classified as “clients” of the school psychologist services and sometimes can even have conflicting opinions and interests. In any case, when dealing with minors, the parents or other legal guardians/tutors must provide informed consent for the assessment and have the right to access any information that is used to make educational decisions about their children (i.e., the assessment results). Regarding sharing information with others, some ethics codes, such as the one by the Portuguese Psychologists Order, determine explicitly that, in the case of children and adolescents under 18 years old, sharing information with other professionals requires authorization from their legal guardians/tutors, even when this assessment is performed in school contexts (OPP, 2021). NASP (2020) also states parental consent is required before sharing sensitive information about a child’s psychological assessment results with third parties. However, there may be exceptions to this rule in situations where there is an immediate threat to the safety of the child or others or when mandated by law or school policy. In such cases, school psychologists may be permitted to share information without parental consent in order to ensure the safety and well-being of the child and those around them.

Therefore, as stated by Fisher (2014), the question that the school psychologists should consider is not “Who is the client?”, but “What are my ethical responsibilities to each of the parties involved?”. Nevertheless, it is important for school psychologists to carefully consider ethical and legal guidelines of their country/context when making decisions about sharing assessment results without parental consent and to document their rationale for doing so. Obtaining informed consent from parents or legal tutors before the assessment, again, is the key, as it should include a “discussion of the limits of confidentiality, who will receive information about assessment or intervention outcomes, and the possible consequences of the assessment/intervention services being offered” (NASP, 2020, pp. 42–43). Thus, psychologists must clearly communicate how the information will be used, who will have access to it, and the steps taken to protect the student’s privacy.

Another related issue is the access to past psychological assessment records, given that in schools this access is frequently needed to inform educational decisions, such as placement in a multitiered system of support. As said before, parents have the right to access the records at any time. Regarding the permission of other professionals to access it, the Portuguese referential does not state explicitly a rule, but the NASP Professional Standards assert that:

To the extent that school psychological records are under their control, school psychologists ensure that only those school personnel who have a legitimate educational interest in a student are given access to that student’s school psychological records without prior parental permission or the permission of an adult student (NASP, 2020, p. 48)

Again, there is the question of ethical responsibilities: to allow the educational professionals to have access to information to make informed decisions, while maintaining the students’ best interest as a priority.

### How to communicate the results?

The assessment results might be communicated to interested parties orally or in the form of a written report. A good report elucidates the rationale and methodology behind the evaluation, detailing the request that led to the psychological assessment. It maintains a balanced perspective by avoiding an exclusive focus on dysfunctions and deficits, thereby mitigating potential negative consequences for the individuals examined. Instead, it should provide guidelines aimed at supporting and empowering the assessed individuals. This is an important aspect that seems to be frequently overlooked. Recent studies in different countries indicate that parents and teachers find that school psychologists’ reports focus more on test results and provide little help in drawing up adequate interventions to support the students’ needs (King et al., 2023; Rahill, 2018). Moreover, the report should clearly outline the conditions and limitations of the psychological assessment conducted and refrain from extensive interpretations or extrapolations that exceed the collected data (Lichtenstein & Ecker, 2019). Only the relevant information must be included in assessment reports, meaning only the necessary information to respond to the request/goal of the assessment (OPP, 2021).

School psychologists must also take into account who is the report recipient. As most of the time those are parents and teachers/educators, the report should, as much as possible, avoid psychological jargon and use an objective, but accessible, language (Walrath et al., 2014). An additional difficulty arises when parents are from linguistically minoritized groups or speak a language which is not the official or dominant one. Is this case, some authors recommend that two copies of the assessment report should be drafted: one in the official language to be presented to the educational professionals and another in the primary language of the parents (Aldalur et al., 2022).

When delivering written reports, the framework for school psychology in Portugal recommends that the psychologists conduct an interview to return the of psychological assessment, at the same time the make the written report available to the recipient (Breia et al., 2024). This procedure aims to reduce the occurrence of misinterpretations and offers the opportunity to the recipient to pose questions about missing or ambiguous information. The same referential recommends that the results of psychological assessment should be communicated, when possible, to both parents (or tutors) and to the child or adolescent, depending on his/her level of maturity (Breia et al., 2024). As stated in the deontological code of the OPP (2021), the communication of assessment results, whether oral or written, should also include an individualized interpretation of the results and an explanation of the limitations of the assessment instruments used. Research clearly points out that the use of digital (online or offline) testing and scoring is now quite prevalent (Benson et al., 2019; Csapó & Molnár, 2019; Dombrowski et al., 2023; Maluf et al., 2022), and usually these instruments produce an automatic (computer-generated) report of results. Returning this report to parents or other recipients, such as teachers, without any other information is not advisable, as misinterpretations can occur (Knauss, 2001).

Psychologists must also take into account that test integrity must be protected, and, therefore, the test materials and protocols should not be disclosed, before or after the assessment, as their use can be invalidated. However, sometimes this is not easy to achieve. Dailor and Jacob (2011) reported that one of the main difficulties of school psychologists was to balance parents’ rights to access psychological assessment protocols while also maintain test materials security. In fact, the most recent version of the NASP professional standards states that

School psychologists respect the right of parents (and eligible students) to inspect, but not necessarily to copy, their child’s (or their own) answers to school psychological test questions, even if those answers are recorded on a test protocol. School psychologists understand that the right of parents (and eligible students) to examine their child’s (or their own) test answers may supersede the interests of test publishers (NASP, 2020, p. 48).

A different matter is related to sending completed test protocols to another psychologist, for example, when parents request it because they want a second opinion outside of the school. In this case, copies of the test protocols can be sent, although it is recommended that these are sent directly to the second psychologist to protect the integrity of the test materials (NASP, 2020; OPP, 2021).

## Conclusion

**Fig. 1:**
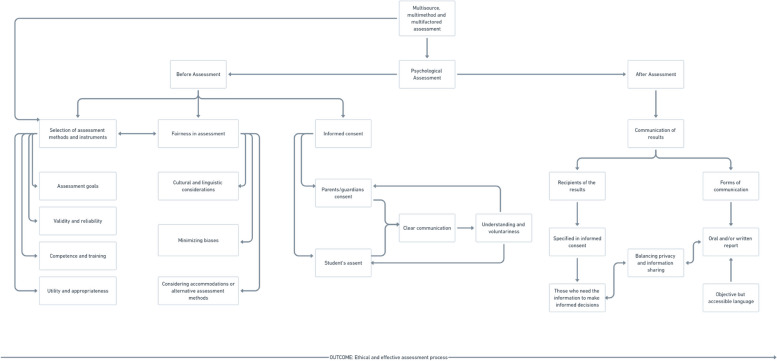
Flowchart depicting ethical issues in assessment, aspects to be considered and recommended strategies

This paper reviews some of the issues encountered during psychological assessments in schools and highlights best practices and recommendations derived from established ethics codes. We focused aspects related to the selection of instruments, ensuring fairness in assessment, and obtaining informed consent, as well as aspects related to the communication of the results after the collection of data, using the chosen assessment methods. Figure 1 presents a flowchart that outlines the critical issues and considerations involved.

The main conclusions can be summarized in four main points. First, the selection of assessment instruments should be guided by validity, reliability, utility, and appropriateness for the specific student population (Canivez, 2019). School psychologists must consider cultural and linguistic diversity to ensure that assessments are fair and unbiased. Second, fostering fairness in assessment involves implementing procedures that are equitable and inclusive. This includes being aware of and addressing any potential biases in test administration and interpretation (APA, 2020). Third, obtaining informed consent requires clear communication with parents or guardians. Psychologists should provide detailed information about the assessment process, its purpose, and how the data will be used, ensuring that consent is both informed and voluntary. Obtaining assent from students is also an important ethical practice, particularly for older children and adolescents who are capable of understanding the assessment process. Collecting assent involves explaining the assessment in a developmentally appropriate manner, ensuring that the student understands what will happen, and obtaining their agreement to participate. This process respects the student’s autonomy and helps build trust, which fosters collaboration. Fourth, communicating the results of the assessments should be handled with sensitivity and confidentiality. Results should be shared with relevant stakeholders, including students, parents, teachers, and other professionals, in a manner that is understandable and constructive.

In conclusion, school psychologists must adhere to ethical guidelines to maintain professionalism and integrity in their work. This includes ongoing professional development and adherence to the ethical standards set by national and international professional organizations professional associations such as the OPP (2021), the NASP (2020) and the ISPA (2021). Addressing these ethical concerns in psychological assessment in school contexts is crucial to promote fairness, accuracy, and the well-being and educational outcomes of students.

## Data Availability

Not applicable.

## Abbreviations

AEA: Association of Educational Assessment – Europe
AERA: American Educational Research Association
APA: American Psychological Association
DIF: Differential Item Functioning
EFPA: European Federation of Psychologists Association
ITC: International Test Commission
ISPA: International School Psychology Association
MTTS: Multi-Tiered Systems of Support
NASP: National Association for School Psychologists
OPP: Order of the Portuguese Psychologists
